# Optimization of biomass and biokinetic constant in Mazut biodegradation by indigenous bacteria BBRC10061

**DOI:** 10.1186/2052-336X-12-98

**Published:** 2014-06-24

**Authors:** Alireza Chackoshian Khorasani, Mansour Mashreghi, Soheila Yaghmaei

**Affiliations:** 1 Department of Chemical and Petroleum Engineering, Sharif University of Technology, Tehran, Iran; 2 Cell and Molecular Research group, Institute of Biotechnology, Ferdowsi University of Mashhad, Mashhad, Iran

**Keywords:** Optimization, Biosystem, Biodegradation, Kinetics, Native bacteria, *Enterobacter cloacae*

## Abstract

Optimization based on appropriate parameters can be applied to improve a process. Mazut degradation as a critical issue in environment requires optimization to be efficiently done. To provide biodegradation conditions, experiments were designed on the least interactions among levels of parameters consisting of pH, Tween 80, glucose, phosphorous source, nitrogen source, and time. Kinetic constants and biomass were calculated based on 16 assays, designed using Taguchi method, which constructed various mazut biodegradation conditions. Kinetics of mazut degradation by newly isolated bacteria *Enterobacter cloacae* closely followed second order kinetic model. Results of the 16 experiments showed that biomass was in the range of 0.019 OD_600_ to 2.75 OD_600_, and biokinetic constant was in the range of 0.2 × 10^-5^ L/ (mg day) to 10^-4^ L/ (mg day). Optimal level for each parameter was obtained through data analysis. For optimal biomass equal to 2.75 OD_600_, optimal pH, Tween80, glucose, phosphorous source, and time were 8.3, 4 g/L, 4 g/L, 9 g/L, and 10 days, respectively. For biokinetic constant equal to 1.2 × 10^-4^ L/ (mg day), optimal pH, Tween80, glucose, phosphorous source, and nitrogen source were 8.3, 1 g/L, 4 g/L, 1 g/L, and 9 g/L, respectively. The optimum levels for biomass and biokinetic constant were the same except the levels of the Tween 80, and phosphorous source. Consequently, mazut may be more degraded with adjusting the conditions on the optimum condition.

## Introduction

Optimization is very prominent to achieve high quality. Each process is optimized based on effective elements. Various parts of process have interaction together. Interactions depend on type and quality of elements, which have to be designed to desirable aims. Then, efficiency of process is assigned by designed condition. There are many methods to design experiments for optimization. They usually organize assays with considering a number of variables and given variable levels. Obtained results are analyzed by statistical methods. Relating diagrams are plotted to help find optimum quantities. Finally, with graphs and analysis of data, optimum conditions are determined [1,2]. Although methods are able to assign optimum quantities, various methods often represent different optimum data for one process. Difference among methods is originated from both their design experiment trends and mathematical directions of attaining optimum data [1]. New methods for optimization have rarely been reported. New optimization models for empty container management [3], and a procedure developed to determine the optimum reaction rate constants in generalized Arrhenius form [4] were reported. Consequently, there is not a general way for optimization.

The Biodegradation of mazut, a source of heavy hydrocarbons, is depended on different ingredients. Several parameters can be effective on hydrocarbon biodegradation, such as oxygen, indigenous microorganisms, nitrogen, phosphor, temperature and pH which can gain the most efficiency with optimization [5-7]. In mazut biodegradation, bioactivity is a significant factor to degrade mazut which has to be optimized. Biokinetic constant is dependent on complex interactions occurred in bacterial medium culture. It is the most main parameter which is investigated for biodegradation. On one hand, bioactivity is directly related to biomass produced. Many studies have already been reported about microbial growth conditions and kinetic parameters. Kinetics investigation was studied on biodegradation of mazut in different mazut concentrations [8,9]. Adopting a new model calculated kinetic and thermodynamic parameters of CA degradation [10]. The effect of fructose concentration on the fungal growth was modeled [11]. The most influential kinetic parameters of a model were estimated to degrade phenol [12]. A mathematical model of the fed-batch reactor has been developed based on mass balance equations of the main process variables such as biomass, glucose, and product; and implemented with kinetic expressions to explain the yeast behaviour [13]. Microbial growth parameters incorporated into mathematical models to predict microbial competition [14]. Biochemically structured models for ethanol fermentation, several aspects of the fermentations were explained by the model [15]. A two-step parameter-estimating strategy was proposed to estimate kinetic parameters pertinent to yeast growth [16]. According to the reports, investigating optimal conditions of biomass and biokinetic constants is so important to achieve higher efficiency for mazut biodegradation.

In this study, we attempted to improve mazut biodegradation via optimization of biomass of *Enterobacter cloacea* newly isolated bacteria BBRC10061, and optimization of kinetic constant of mazut biodegradation obtained based on appropriate experiments designed. In the assays, we used five variables for optimization of biomass and biokinetic constant. They are followed as pH, Tween 80, glucose, phosphorous source, nitrogen source (only for kinetic constant), and time (only for biomass). Finally, optimal conditions for biomass and kinetic constant were computed by ANOVA.

## Materials and methods

### Microorganism

*E. cloacae* BBRC10061 is indigenous bacteria strain isolated from oil-contaminated soil where buses were fixed and fuelled in Mashhad, Iran. It was procured from the biochemical and bioenvironmental research center (BBRC) that was a local culture collection in Sharif University of Technology; Tehran, Iran. The microorganism was maintained in glycerol stock at -20°C for further use.

### Experimental design and condition

A well known experimental design technique, namely L16 orthogonal array (OA) design by Taguchi method, was employed to investigate the effect of significant parameters on bacterial growth and kinetic constant in biodegradation of mazut. The 5 parameters having 4 levels were selected (Table 1). From them, time and nitrogen source didn’t used to optimize kinetic constant and biomass respectively; in this case, time to optimize kinetic constant was all of the ten-day period, and nitrogen source to optimize biomass was 9 g/L of (NH4)_2_SO4. According to the Table 1, the 16 experiments were designed based on the least interactions among variables levels. All experiments were conducted in duplicate in this study.

**Table 1:** **L**_**16**_**-Orthogonal array design for biomass and kinetic constant; their experimental and optimization results**

**Experiment no.**	**Level of parameter**							
	**pH**	**Tween80 (g/L)**	**Glucose (g/L)**	**KH**_**2**_**PO**_**4**_ **+ K**_**2**_**HPO**_**4 **_**(g/L)**	**(NH**_**4**_**)**_**2**_**SO**_4_^** ^**(g/L)**	Time^* ^**(day)**	**Biomass (OD**_**600**_**)**	**K × 10**^**5 **^**(mg**^**-1**^ **L day**^**-1**^**)**
1	5.8	0	0	1	1	2	0.019	4
2	6.8	1	1	3	3	4	0.736	1
3	7.3	2	2	5	5	8	1.59	2
4	8.3	4	4	9	9	10	2.75	5
5	5.8	1	2	9	1	8	0.992	8
6	6.8	2	4	1	3	10	1.034	4
7	7.3	4	0	3	5	2	1.094	2
8	8.3	0	1	5	9	4	0.71	6
9	5.8	2	1	9	5	10	1.153	3
10	6.8	4	2	1	9	2	0.785	7
11	7.3	0	4	3	1	4	0.851	4
12	8.3	1	0	5	3	8	0.514	5
13	5.8	4	1	5	1	2	1.58	0.2
14	6.8	0	2	9	3	4	1.002	4
15	7.3	1	4	1	5	8	1.426	10
16	8.3	2	0	3	9	10	1.379	10
Optimization of kinetic constant	8.3	1	4	1	9	-	-	12
Optimization of biomass	8.3	4	4	9	-	10	2.75	-

^*^This parameter just used to optimize biomass.

^**^This parameter just used to optimize kinetic constant.

A mineral salt medium was used which consisted of composition per liter: MgSO_4_.7H_2_O 0.1 g, CaCl_2_ 0.01 g, FeSO_4_.7H_2_O 0.01 g. The medium was distributed into 50 mL centrifuge tubes, and experiments mediums were completed based on the Table 1. In every experiment, 2000 ppm concentration (0.1 g per 50 mL) of mazut, purchased from NIOPDC (National Iranian Oil Products Distribution Company), was added into each test tube containing medium autoclaved at 121°C for 20 min. Prepared blank for each experiment contained all of the components except mazut. Then, bacterial inocula were added into the mediums according Table 1. For 16 designed experiments, five subcultures were prepared since investigation was plotted for 5 steps in 5 different time periods. All subcultures were shaker incubated at 33°C, 160 rpm for 2, 4, 6, 8 and 10 days.

### Analytical methods

Mazut concentration was measured by spectrophotometer (S2000 UV/VIS). To extract mazut from medium, 3 mL chloroform was added into medium and centrifuge tube was shaken and blended till all mazut was dissolved in chloroform. Two separate phases created contained mazut and chloroform in under, and mineral phase in up respectively. Mineral part voided of mazut, and other organic components was decanted to extract organic phase. After that, organic phase was sampled and diluted by chloroform solvent. Absorbance of mazut in medium was measured at 450 nm by spectrophotometer. Sample concentration was estimated based on absorbance-concentration curve. Also absorbance of samples at 600 nm recorded as optical density (OD_600_) was used for biomass measurement. pH was estimated by pH meter (3020 model, Jenway) with sampling culture medium.

### Evaluation of kinetic constant

The mazut concentrations recorded from 5 time steps were tested to first, second, and third order kinetic models. Ln (*C*_*z*_), 1/*C*_*z*_, and 1/(*C*_*z*_)^2^ versus t, where *C*_*z*_ and t are mazut concentration (ppm) and time (day), respectively; resulted the biokinetic constant (*K*) for first, second, and third order models, respectively.

### Analysis of data

For each of the experiments, kinetic constant and biomass were evaluated in the Table 1. Based on the results and levels of the parameters, ANOVA was used to determine optimum data; also, main effects plots for biomass and biokinetic constant were drawn to ascertain optimum level. All of the calculations and plots were done using Minitab16.

## Results and discussion

16 experiments designed were executed. According to the Table 1, biomass recorded ranged from 0.019 to 2.75, which demonstrates different impacts of various formulations of medium. Interaction among specific amounts of each element achieves unique biomass concentration.

**Figure 1:**
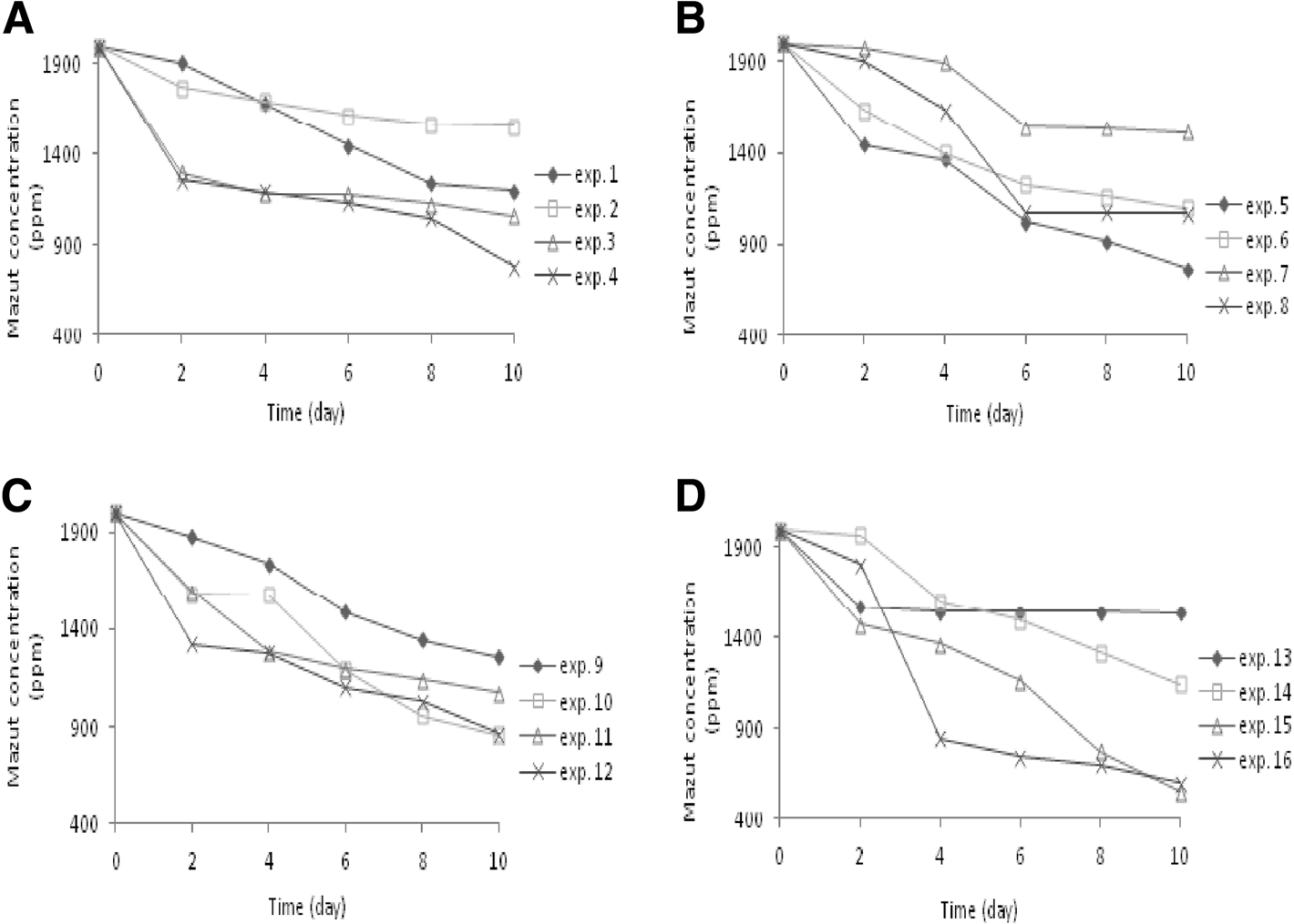
Mazut concentrations in period of 10 days for (A) assays of 1–4, (B) assays of 5–8, (C) assays of 9–12, and (D) assays of 13–16.

Mazut concentrations obtained from the experiments were investigated by order kinetic models. Investigations showed that biodegradation of mazut followed second order kinetic model more closely. However, concentration trends versus time depicted on Figure 1, relate different orders but in this case, generally second order was more precisely to estimate kinetic constants for all of the assays. Kinetic study showed that the data of asphaltene biodegradation fitted to Tessier model [17]. Thus, type of process specifies appropriate kinetic model to data fitting.

In the Table 1, calculated biokinetic constants assigned assortment from 0.2 × 10^-5^ L/ (mg day) to 10^-4^ L/ (mg day). This rather wide range was referred to the media formulations. The kinetic constant was depended on enzymes produced by *E.cloacae*. Enzyme efficiency relied on ingredients and conditions. Therefore, all of the parameters selected to the optimization were effective on mazut degradation. The optimal value of kinetic constant average for asphaltene biodegradation was 0.138 g/ (L day) for *B. lentus*[17]. The optimal biokinetic constant of *Pseudomonas putida* was 0.16 g/ (L day) for the biodegradation of TNT [18]. The motor oil biodegradation capacity of *Pseudomonas aeruginosa* was enhanced from 0.178 g/ (L day) to 0.34 g/ (L day) in optimum conditions [19]. Thus, compared with the data obtained from this study, 10^-4^ L/ (mg day) that is exchanged into 10 g/ (L day), *E. cloacae* BBRC10061 is more efficient than other pure bacteria reported for biodegradation of hydrocarbons.

**Figure 2:**
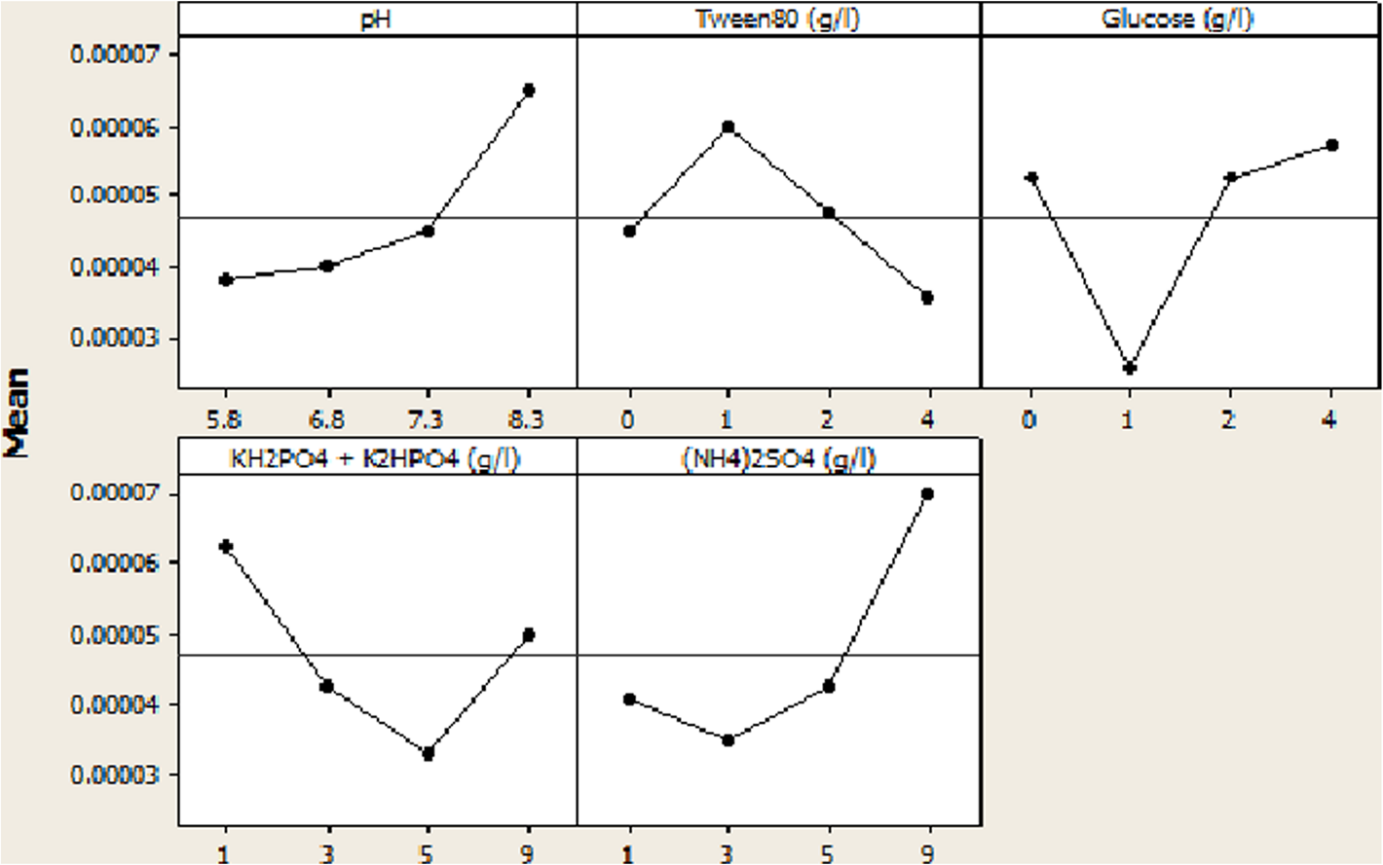
Main effects plot of the parameters for biokinetic constant.

**Figure 3:**
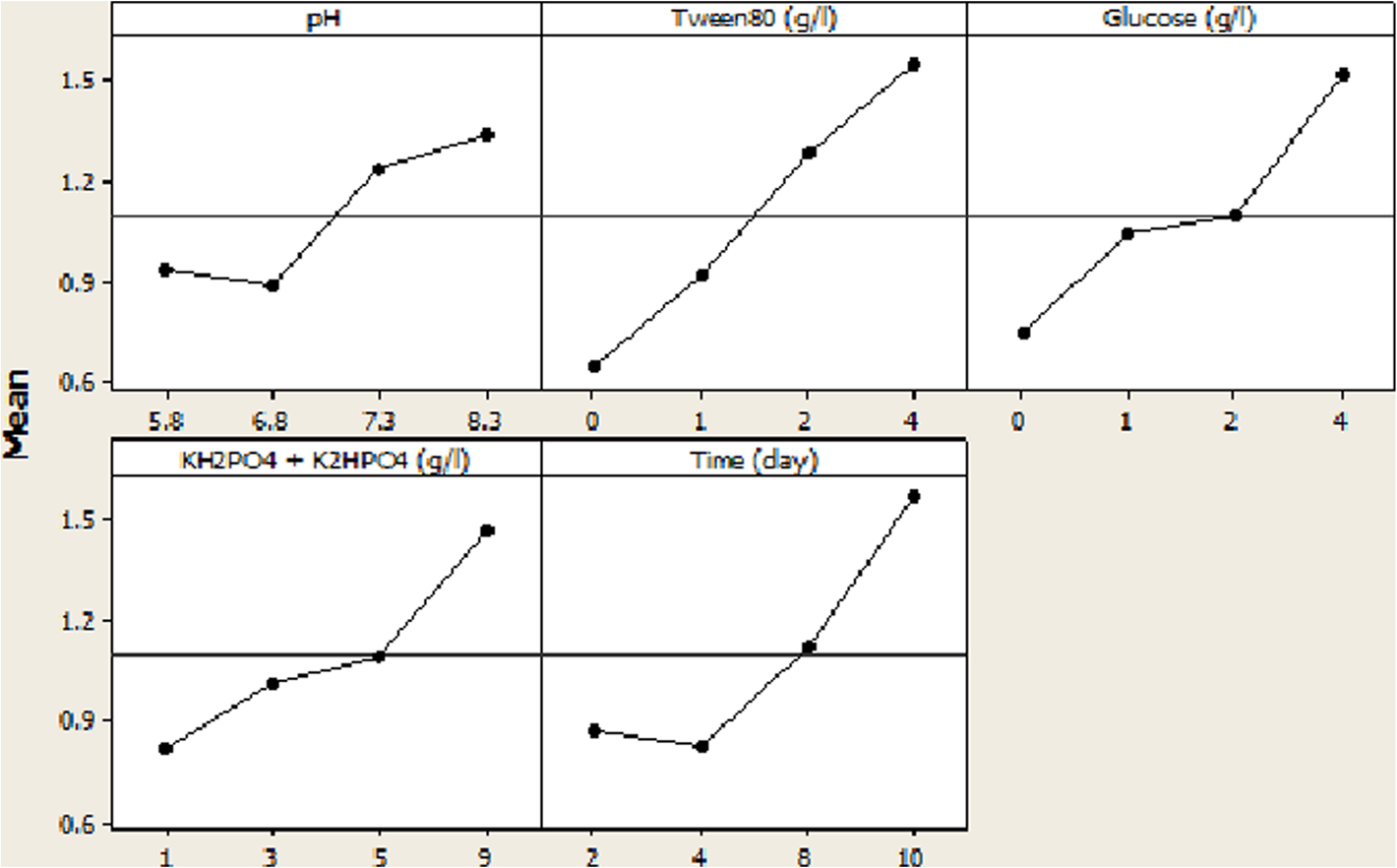
Main effects plot of the parameters for biomass.

Results specified whatever biomass grew more, enzyme production and consequently biokinetic constant were not raised by biomass; because enzyme production was performed in special period of microbial growth. According to the Figures 2 and 3, ANOVA calculated parameters levels for optimum conditions of biomass and biokinetic constant. *E. cloacae* BBRC10061 to degrade mazut needed basic medium so that pH at 8.3 yielded bacterial growth and bioactivity better. Also, the results demonstrated enzymes produced by the bacteria were performed at basic environments, classified into basic enzymes. A consortium of the mixed facultative anaerobes *Acinetobacter calcoaceticus* and *Pantoea agglomerans* highly degraded LAS and SDS at pH 8.5 [20]. Optimal pH in various processes were 6 for biodegradation of 4-chlorophenol by *Candida tropicalis* PHB5 [21], 6.7 for asphaltene biodegradation by pure cultures of *B. lentus*[17], and 7 for the biodegradation of TNT by *Pseudomonas putida*[18]. The optimal growth and biomass yields occurred at pH 6.8 in chemostat [22]. The optimum condition for growth and phenol degradation of *P. putida* was as follow pH 7.0 [23]. Thus, type of microorganism degrading specifies pH in the medium. BBRC10061 in this study can efficiently degrade in basic medium, but related to the other reports; neutral and acidic mediums were used to effective biodegradation based on microorganism.

Although Tween80 as a surfactant increased solubility of the mazut, it could be consumed as a carbon source [20]. Extra supplement of Tween80 inhibited production of biosurfactant and was degraded to bacterial consumption. The high bacterial growth required more carbon sources, but high degradation rate needed biosurfactant production. Therefore, increasing Tween80 concentration to 4 g/L caused more biomass production because of the carbon source added, and simultaneously inhibition of biosurfactant production. Thus, decreasing Tween 80 to 1 g/L could force the bacteria to produce more biosurfactant instead of surfactant (Tween80) and more raise biokinetic constant consequently because microorganism efficiently performed with the bioingredients naturally. LAS (700 ppm) and SDS (8000 ppm) as surfactants at higher concentrations became toxic to the *A. calcoaceticus*[20]. The optimal tween80 concentration for the biodegradation of TNT by *Pseudomonas putida* was 0.1% [18]. Thus, according to microorganism and substrate, amount and type of a surfactant can be changed into produce biomass or raise kinetic constant.

The bacteria needed glucose to metabolism. Thus, increasing glucose concentration to 4 g/L, this was the most concentration, yielded bacterial ability to produce enzymes and grow biomass more. Biodegradation of LAS was accomplished by additional supplementation of some carbon sources such as glucose, sucrose, and maltose [20]. The optimal carbon source for phenol degradation of *P. putida* was glucose at 0.34 g/L [23]. Therefore, microbial needs for the metabolism in presence of objective substrate determine type and concentration of carbon source.

In this study, rate of degradation depended on the least concentration of phosphorous source (1 g/L). In contrast, the bacteria consumed the most concentration of phosphorous source (9 g/L), which probably was to produce biomaterials except enzymes such as nucleic acids. Optimal phosphor (0.14 g) found by design expert for the bioremediation of weathered crude oil (2 g) [24]. Optimal phosphate was 0.6 g/L for biodegradation of 4-chlorophenol by *Candida tropicalis* PHB5 [21]. Optimal phosphor was supplemented with 12.71 mg/L for biodegradation of crude oil [25]. Optimal NaH_2_PO_4_ for BTEX degradation was found as 0.07 g/L [26]. The optimal medium of phosphorous source (K_2_HPO_4_) was 4.049 g/L in biodegradation of motor oil by indigenous *Pseudomonas aeruginosa*[19]*.* The optimum phosphorous composition was determined to be Na_2_HPO4 2.75 g/L for diesel oil degradation by *Rhodococcus erythropolis*[27]. Thus, the least phosphorous composition is essential for metabolism. Not only phosphorous concentration depends on microorganisms and objective materials which are to degrade, but also it has not to inhibit microbial growth. Therefore, optimum concentration of phosphorous source can be tuned based on microbial process.

According to the production of proteins which require high concentration of nitrogen, *E. cloacae* engrossed the most concentration of nitrogen source (9 g/L) to increase degradation and growth rates. Minerals are necessary to microbial metabolisms. Also, extra amounts of the minerals can inhibit microbial metabolisms. Supplementation with nitrogen nutrients such as casein and tryptone has increased the biodegradation extent of LAS from 60% to 90% [20]. Optimum nitrogen determined to biodegradation of weathered crude oil (2 g) was 0.68 g [24]. Optimum yeast extract as nitrogen source for biodegradation of 4-chlorophenol by *Candida tropicalis* PHB5 was obtained 1.5 g/l [21]. For biodegradation of crude oil concentration of 1 g/L, optimal nitrogen was supplemented with 190.21 mg/L [25]. The optimum NH_4_Cl as a nitrogen source was 0.025% for the biodegradation of TNT by *Pseudomonas putida*[18]. Optimum NH_4_Cl for BTEX degradation was found as 1.484 g/L [26]. The maximum removal of phenol by *Candida tropicalis* Z-04 was obtained under the optimum conditions of yeast extract 0.41 g/L [28]. The optimum nitrogen conditions for growth and phenol degradation of *P. putida* were yeast extract at 0.16 g/L and ammonium sulfate at 2.32 g/L [23]. The optimum nitrogen nutrient amendment composition were determined to be (NH4)_2_SO_4_ at 2.53 g/L and 2.05 g/L, and yeast extract at 10.19 mg/L and 14 mg/L, for diesel oil degradation by *Rhodococcus erythropolis* and *Acinetobacter beijerinckii ZRS* respectively [27,29]. Thus, optimum extent of nitrogen source is completely related to microbial metabolism. Also, source of nitrogen such as ammonium source or yeast extract are facilitative to digest for microorganisms.

Optimal time for the bacterial growth was 10 days. According to Figure 3, at the early time, the bacteria occupied to adapt themselves to the medium. After that, the growth was satisfyingly raised from extent of 0.019 OD_600_ to extent of 2.75 OD_600_ because of microbial adaptation. Optimal incubation time for Enhanced biodegradation of 4-chlorophenol by *Candida tropicalis* PHB5 was calculated 60 hours using Taguchi [21]. The optimum biomass for asphaltene biodegradation reached to 2.3 g/L [17]. Thus, growth time based on objective bioprocess can be changed for each microorganism. In general, bioprocess of degradation of hard-degradable components has a long time of growth.

It is demonstrated that to achieve high degree degradation, proper medium prepared to high biomass is not obligatorily to gain high kinetic activity. On the other hand, using optimum condition for microbial growth can’t warrant kinetic bioactivity that should be to degrade materials in biodegradation.

According to the reports, optimization of biomass and kinetic constant has not been investigated for biodegradation of mazut. Thus, comparing the data of this study with other reports data is not easy. The levels of the parameters reported in this study can be able to optimize not only mazut biodegradation but also degradation of other heavy hydrocarbons, if the strain BBRC10061 hires to degradation.

## Conclusion

Biokinetic constant and biomass were optimized with the proper factors which affected on biodegradation of mazut. ANOVA represented optimum data and main effects plots of the each parameter. There were accordances among optimum levels of the parameters for biomass and biokinetic constant, but all of the bacterial growth and bioactivity needs were not the same. Therefore, whatever causing microbial growth efficiently was not similarly able to yield high bioactivity to degrade hydrocarbons.

## Competing interests

The authors declare that they have no competing interests.

## Authors’ contributions

ACK, carried out this study as part of his M.Sc. project, planed the original research of the project, and wrote the manuscript. MM, supervised the microbial part of this research, and revised the manuscript. SY, supervised the mathematical part of the project, and read the manuscript. All authors read and approved the final manuscript.
